# Analysis of Dynamic Plantar Pressure before and after the Occurrence of Neurogenic Intermittent Claudication in Patients with Lumbar Spinal Stenosis: An Observational Study

**DOI:** 10.1155/2020/5043583

**Published:** 2020-07-01

**Authors:** Wei Wei, Chao Xu, Xiao-Jiang Yang, Chang-Bo Lu, Wei Lei, Yang Zhang

**Affiliations:** Department of orthopedics, Xijing Hospital, The Airforce Military Medical University, 710032, China

## Abstract

Lumbar spinal stenosis (LSS) is a common disease in the elderly population; it has been reported that patients with LSS have an abnormal gait pattern due to symptom such as neurogenic intermittent claudication (NIC); however, no detailed reports exist on the plantar pressure distributions in LSS patients with NIC. To analysis the plantar pressure characteristics of LSS patients, the Footscan® pressure system was used to perform dynamic plantar pressure measurements in 20 LSS patients (age, 69.5 ± 7.2 years) before and after the occurrence of NIC. The contact time (CT), foot progression angle (FPA), pressure-time integral (PTI), and contact area (CA) were collected and compared between the LSS patients and age-matched healthy subjects in each measurement. The LSS group showed an increase in forefoot CT%, PTI, and CA% in both measurements compared with those in the control group. After the occurrence of NIC in the LSS group, CT%, PTI, and CA% of the forefoot increased further compared with those before the occurrence of NIC. In addition, after the occurrence of NIC, the PTI and CA% of the forefoot shifted from the medial foot to the lateral foot. The results suggested that the plantar pressure distributions of patients with LSS differs from normal subjects due to the posture of waking with lumbar forward flexion, and the forefoot bears a higher relative load. In addition, the occurrence of NIC could affect the plantar pressure distribution of the patients with LSS, predicting the patient's risk of falling to the anterior direction and to the symptomatic side.

## 1. Introduction

Lumbar spinal stenosis (LSS) refers to the nerve compression syndrome, in which the spinal canal narrows and compresses the dural sac, spinal cord, or nerve root [1]. The prevalence of LSS increases with age; it is about 9.3% in the general population and up to 47% in individuals older than 60 years [2, 3]. For individuals older than 65 years, LSS can be the most common cause of lumbar surgery [4]. The incidence of LSS has increased because of the burden of aging population. Patients with LSS may cause negligible economic problems considering their limited daily activities and weakened labor capacity [4, 5]. Therefore, clinicians are focusing more on the diagnosis and treatment of LSS. Magnetic resonance imaging (MRI) technology is often the first choice for LSS due to its superior soft tissue resolution [6]. However, the degree of LSS presented by imaging and the severity of clinical symptoms may have a relatively low correlation. Although some individuals do have anatomical LSS, they may not have relevant clinical symptoms [7]. Therefore, besides imaging data, clinicians should consider the medical records and physical signs of the patients at the same time for the diagnosis of LSS [7, 8].

Patients with LSS usually seek medical treatment because of walking disorders. The poor walking ability indicates severe compression of the spinal cord and nerve. Therefore, assessing the patient's walking function is essential for the diagnosis and treatment of LSS. Many recent studies investigated the gait patterns of patients with LSS. The findings revealed that the walking patterns of patients with LSS were different from those of ordinary subjects, mainly manifested by wide-based gait, increased gait variability, and balance disturbances [9–11]. In addition, studies have shown that the balance of patients with LSS could get worse after the occurrence of neurogenic intermittent claudication (NIC), leading to a higher risk of falling [11]. However, many gaps still remain in the study of mode changes in gait patterns before and after the occurrence of NIC. Specific mechanisms of gait changes need to be elucidated.

The changes in gait can probably affect the pressure distribution under the foot [12]. As an important substitution of gait analysis, plantar pressure analysis is the basis for analyzing and measuring abnormal plantar pressure distribution and gait. It has significance for the etiology analysis, diagnosis, function, and treatment evaluation of diseases related to walking disorders [13]. However, none of the studies have analyzed the pattern of plantar pressure distribution in patients with LSS or the effect of NIC on the distribution of plantar pressure. This study used the Footscan® pressure system to perform dynamic plantar pressure tests on patients with LSS. The aim of this study was to reveal the changes in the distribution of plantar pressure and the risk of falling before and after the occurrence of NIC in patients with LSS. The differences in plantar pressure distributions and the mechanism of their occurrence should be explained to provide theoretical support and data reference for the diagnosis and treatment of patients with LSS.

## 2. Methods

### 2.1. Participants

This observational study is a cross-sectional, uncontrolled intervention. From October 2017 to April 2019, patients with LSS in our institution were routinely analyzed for plantar pressure after admission to improve the database of plantar pressure for patients with LSS. The database was reviewed, and the case group was selected. The inclusion criteria were as follows: (1) L4–L5 level spinal stenosis on imaging examination [11, 14] and a history of intermittent claudication; (2) symptom onset within 1 year with no history of invasive treatment; and (3) no history of lower extremity trauma in the last 1 year. Subjects were excluded if they: (1) had spinal stenosis in other levels of the spine; (2) had spine-related diseases, such as scoliosis, lumbar spondylolisthesis, spinal trauma, and history of surgery; (3) had diseases that could affect walking function, such as neuromusculoskeletal, vestibular, cardiopulmonary disorders, and vascular lesions of the lower limbs, hip, knee, and ankle joints; and (4) could not walk without aid. A group of healthy subjects of the same age was recruited as the control group. Subjects in the control group had no previous history of trauma, surgery, or motor system diseases. The characteristics of participants were collected for comparison, including age, height, weight, foot length, body mass index (BMI), and the T-score of the body mineral density. This study was approved by the Medical Ethics Committee of Xijing Hospital, Air Force Military Medical University, China. Informed consent for the plantar pressure test and data application was obtained from the participants or their immediate relatives.

### 2.2. Instrumentation and Pedobarographic Analysis

**Figure 1:**
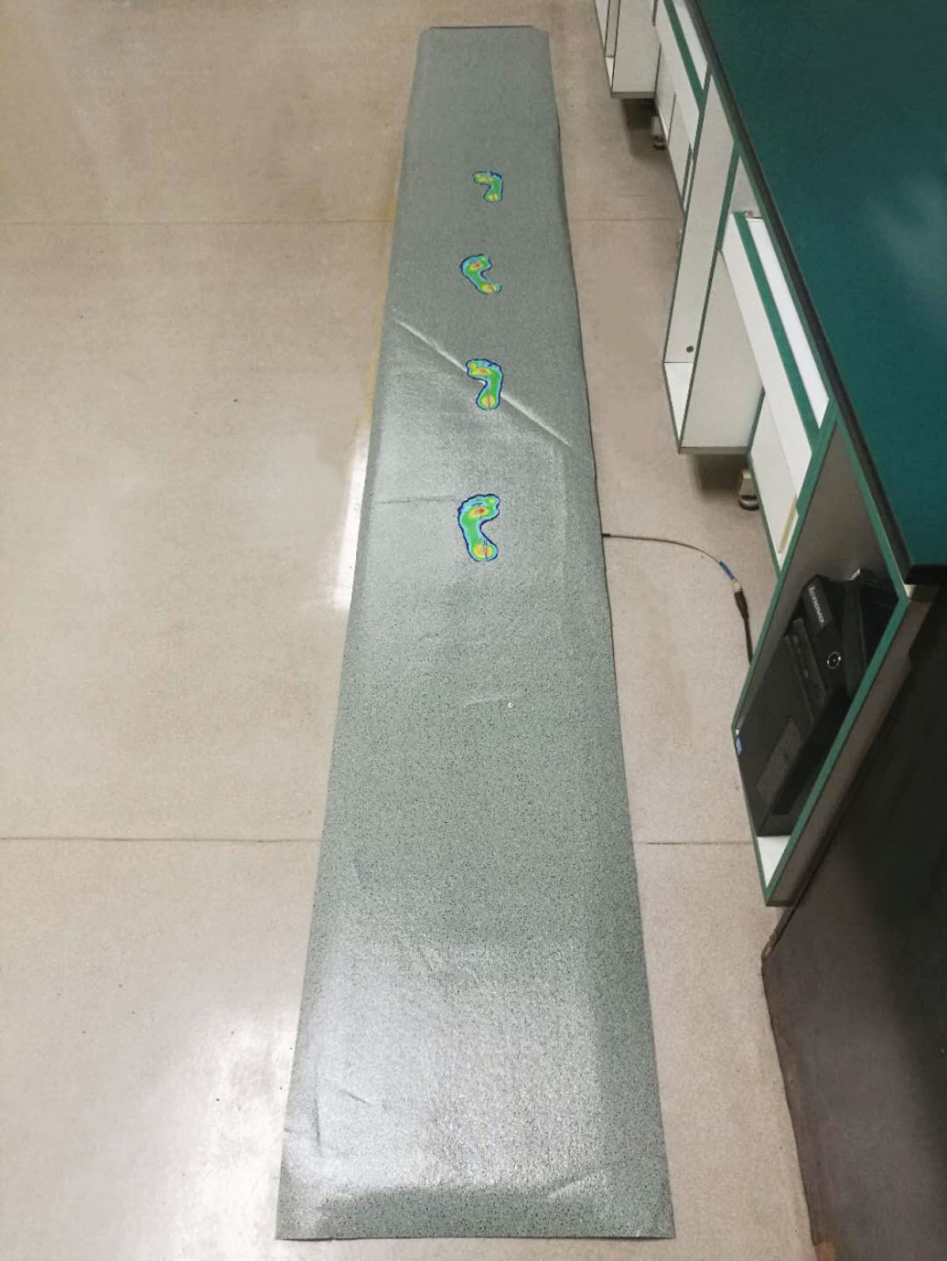
Schematic diagram for walking area of plantar pressure analysis system.

Data were collected using the Footscan® 3D pressure system (RSscan International, Belgium), which has been proved to have good reliability and repeatability [15]. The size of the force plate was 2 × 0.4 × 0.02 m3, with 16,384 sensors distributed. The minimum measurement area was 0.25 cm^2^, and the sampling frequency was 125 Hz. Before each measurement, the test system was calibrated. The force plate was located at the center of a carpet with the same external dimension to provide a “complete platform,” 4 m in length, to ensure that a minimum of three steps was taken before data collection. The platform was disguised with a thin liner of EVA (ethylene-vinyl acetate copolymer) material to avoid targeting effect [15] (Figure 1). The Footscan® system was calibrated before each measurement session following the manufacturer's protocol, and all the participants initially completed 2-min acclimatization walking trails along the measuring platform. The plantar pressure data of each participant were measured using two protocols. For the LSS group, the first measurement was performed after resting for 2 min, and the second measurement was performed after continuous walking until the symptoms of NIC occurred. The control group took the measurements after resting for 2 min and after walking for 5 min. During the measurement, each participant was asked to perform the tests barefoot at their comfortable walking pace, looking forward. In each measurement, about 3-4 consecutive steps were captured, only one representative step (complete footprints, heel-strike pattern, and no obvious adjustment) was artificially selected for analysis and processing. For the LSS patients, the symptomatic side or the more severe side was selected; for the control group, the dominant foot was selected [11]. The aforementioned measurement steps were repeated until three sets of valid data were collected.

**Figure 2:**
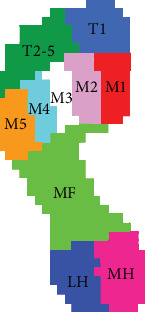
Schematic diagram for 10 partition zones of the foot. T1: hallux. T2–5: toes 2–5. M1: first metatarsal. M2: second metatarsal. M3: third metatarsal. M4: fourth metatarsal. M5: fifth metatarsal. MF: midfoot. LH: lateral heel. MH: medial heel.

The Scientific Footscan® software automatically divided the foot into the following 10 areas according to the anatomical structure of the foot (Figure 2): toe 1 (T1), toe 2–toe 5 (T2–T5), metatarsal 1–metatarsal 5 (M1–M5), midfoot (MF), medial heel (MH), and lateral heel (LH). In addition, to facilitate the overall comparison, the foot was divided into three parts based on the front and back edges of the MF: forefoot (T1, T2–T5, and M1–M5), MF, and heel (MH and LH). The longitudinal axis of the foot divided the anterior and posterior feet into two parts: the medial foot (T1, M1, M2, and MH) and the lateral foot (T2–T5, M3–M5, and LH). As the symptomatic side of the patient with LSS was selected for analysis, the lateral side of the foot corresponded to the symptomatic side.

**Figure 3:**
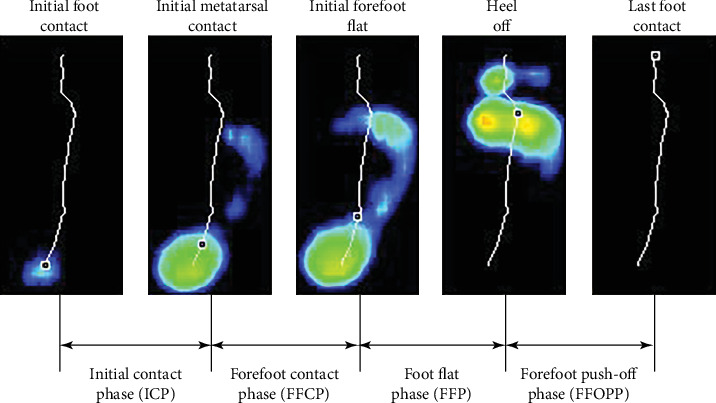
Schematic diagram for 4 subphases of the stance phase.

At the same time, the software divided the entire stance phase of the foot into the following four phases according to the sequence of the ground contact time (CT) of each anatomical structure: (1) initial contact phase (ICP), (2) forefoot contact phase (FFCP), (3) foot flat phase (FFP), and (4) forefoot push-off phase (FFPOP) (Figure 3).

### 2.3. Data Analysis

The following plantar pressure parameters were collected using the software: CT in each subphase, contact area (CA) in each zone, pressure-time integral (PTI) in each zone, and foot progression angle (FPA). Among these, CA was corrected by the proportion of each area in the total plantar area (CA%), and CT was corrected by the proportion of each subphase in the total standing phase of one foot (CT%). The average of each parameter in the three valid data was used as the analysis data of each participant. The data were divided into four groups: first measurement in the LSS group, second measurement in the LSS group, first measurement in the control group, and second measurement in the control group.

### 2.4. Statistical Analysis

The sample size was determined by a pilot study (6 subjects; LSS group, *n* = 3; control group, *n* = 3). The G∗power software (Version 3.1.9.6) was used to calculate the required sample size affording a significance level of 0.05, a power of 0.95, and an effect size of 0.65 (calculated using the means ± standard deviations of data during the pilot study). The required sample size is 28 subjects (LSS group, *n* = 14; control group, *n* = 14).

The data were entered into SPSS 23.0 software, and the Kolmogorov–Smirnov single-sample test and scatter plot test were used. The experimental data conformed to the normal distribution. Therefore, the differences between FPA, CA%, CT%, and PTI in the two measurements of the LSS group and the two measurements of the control group were analyzed using the paired *t*-test. Differences between the LSS group and the control group in the first and second measurements were analyzed using the independent-samples *t*-test, and the differences were statistically significant when *P* < 0.05.

## 3. Result

20 patients with LSS (12 males and 8 females) were included in the experimental group (LSS group), aged 56–73 years, with an average age of (69.5 ± 7.2) years. Five cases of relatively narrow L4–L5 level were found on imaging 15 cases with absolute stenosis [6]. 20 age-matched healthy control subjects were recruited from the community surrounding the hospital. No significant differences in age, height, weight, foot length, BMI, and T-score were observed between the LSS group and the control group (*P* > 0.05; Table 1).

The comparison results of CT% and total CT in the stance phase of each group are shown in Table 2. Compared with the first measurement results of the LSS group, the CT% of ICP decreased and the CT% of FFPOP increased. The CT% of ICP and FFCP was smaller and that of FFP and FFPOP increased in the LSS group compared with the second measurement results of the control group. Compared with the first measurement in the LSS group, the ICP and FFCP decreased and the proportion of FFP and FFPOP increased. In terms of total CT, the two measurements in the LSS group significantly increased compared with those of the control group, and the second measurement in the LSS group significantly increased compared with the first measurement. No significant differences in CT% and total CT were observed between the two measurements in the control group.

Table 3 presents the comparison of the FPA of the two measurements in each group. The FPA in the LSS group increased significantly relative to the two measurements in the control group. No significant difference was found between the two measurements in the LSS group and the control group.

The comparison results of the PTI in each region of the plantar with two measurements are summarized in Table 4. No significant differences in PTI were observed in the control group between the two measurements. The first measurement results in the LSS group showed an increase in PTI in the M1–M2 region compared with the control group; the second measurement results showed a significant increase in the PTI in the M1–M4 region compared with the control group; and the PTI in the MH and LH regions significantly decreased. The second measurement in the LSS group significantly increased the PTI in the first M1–M4 area, and the PTI in the MH and LH areas decreased significantly.

Regarding the proportion of CA in each area, the control group had no statistically significant difference between the two measurements. Compared with the control group, the CA% in the first measurement results of the LSS group in the M1 and M2 areas increased. In the second measurement results, the CA% significantly increased in the T1, T2–T5, and M1–M4 areas and significantly reduced in the MH and LH areas. The comparison of the CA% of the two measurements in the LSS group showed that the results of the second measurement presented a significant increase in the CA% of the T1, T2–T5, and M1–M4 regions and a significant decrease in the CA% of the MH and LH regions (Table 5).

Overall, the LSS group showed an increase in forefoot CT%, PTI, and CA% in both measurements compared with those in the control group. After the occurrence of NIC in the LSS group, CT%, PTI, and CA% of the forefoot increased further compared with those before the occurrence of NIC. In addition, after the occurrence of NIC symptoms, the PTI and CA% of the forefoot shifted from the medial foot to the lateral foot.

## 4. Discussion

LSS is a common and disabling disease in the population over the age of 60 [1]. The pathological process is mainly the degeneration and overgrowth of the bones, ligaments, and synovial tissue that constitute the lumbar spinal canal. This gradually leads to the compression of nerves and blood vessels in the spinal canal, or prolapse into the spinal canal due to disc degeneration resulting in a series of clinical symptoms [1, 2, 14]. The complaints of patients with LSS are generally not typical; they can be pain or numbness and weakness in the lower back and buttocks, presenting different degrees of NIC [14, 16]. Although MRI and other imaging examinations can objectively present the state of the lumbar spinal canal and anatomically observe LSS, they cannot reflect the severity of the patient's symptoms [13], and hence are not enough to accurately diagnose LSS clinically. As the main symptom of LSS [16], NIC is usually found when the patient is walking, making the diagnosis more challenging. Gait analysis, as an emerging detection method, provides a new insight for objectively assessing the walking ability of patients with LSS. However, the parameters for gait and performance of LSS have not been fully defined. Previous studies also focused on the kinematic and kinetic parameters of lower limb joints [17–19]. In this study, the Footscan® pressure system was used to perform a dynamic plantar pressure test on patients with LSS, and the changes in plantar pressure distribution before and after the occurrence of NIC and their differences against those of healthy controls were compared. The plantar pressure distribution of patients with LSS was evaluated objectively, which could better explain the abnormal performance of patients with LSS in kinetics and kinematics, providing an important reference for the application of plantar pressure analysis in LSS.

In the gait analysis study of patients with LSS by Garbelotti et al. [20], patients with LSS had shorter stride length, slower walking speed, and significantly longer stance phase compared with healthy subjects, which could be taken as the compensation strategy. Although the parameters, such as stride length and walking speed, were not directly measured in this study, the results showed that after the occurrence of NIC, the total CT in the LSS group was significantly longer than that in the control group, and no significant difference was found before the occurrence of NIC. This showed that the occurrence of NIC in patients with LSS could result in a decrease in their balance function, causing the patients' requirement of more time to stabilize the body before entering the next gait cycle [10, 20, 21]. Further, the time distribution of each subphase was examined, and the first measurement of the ICP in the LSS group was found to be smaller than that in the control group and the FFPOP was larger. After the occurrence of NIC in the LSS group, ICP and FFCP decreased significantly and FFP and FFPOP increased significantly. Patients with LSS tended to keep the lumbar forward and flexion walking to reduce the symptoms of nerve root compression [22]. This gait pattern could cause the center of gravity to move in the anterior direction [23]. Hence, the patients needed to transfer the center of pressure from the hindfoot to the forefoot faster, resulting in an increase in the CT of the forefoot. In general, in the LSS group, the proportion of the forefoot area increased and the proportion of the hindfoot area decreased in each subphase time distribution compared with the control group before the occurrence of NIC. This characteristic was more pronounced after the occurrence of NIC, indicating that patients with LSS would maintain mild lumbar forward flexion at the beginning of walking. After the symptoms of lower limbs caused by continuous walking, the patient's lumbar spine was further forward flexed, the center of gravity shifted more to the anterior direction, and the transfer of loading was faster to reduce symptoms. These results showed that patients with LSS had an anterior-posterior balance disorder, and they might have a higher risk of falling at the same time [23].

In this study, irrespective of the occurrence of NIC, the FPA was larger in the LSS group compared with the control group, and the foot was more externally rotated when walking. FPA referred to the angle between the longitudinal axis of one foot and the walking direction, and its outward rotation indicated that the patient had an “out-toe” gait when walking [24]. Previous studies confirmed that patients with LSS had a wide-based gait [17], which was mainly manifested by awkward and faltering walking patterns. These patterns could reflect the characteristics of patients with unstable trunk and limited dynamic balance [11]. In addition, a large number of studies showed that LSS could affect the balance function of patients [25]. Therefore, it was hypothesized that patients in the LSS group walked in the “out-toe” gait to obtain a wider base and thus maintain balance, so as to reduce the swing from one side to another [26].

Marte et al. [27] and Lai et al. [28] showed that the changes in FPA could affect the distribution of plantar pressure. When FPA was externally rotated, it promoted the transfer of plantar pressure to the forefoot and medial feet. The findings of the present study were consistent with this conclusion to some extent. Before the occurrence of NIC in the LSS group, PTI and CA% increased relative to the M1–M2 area in the control group, showing the transfer of pressure to the forefoot and medial feet. However, after the occurrence of NIC in the LSS group, the forefoot loading and the CA increased further. At the same time, the hindfoot loading decreased and the CA decreased. However, no significant difference in FPA before and after the occurrence of NIC was observed in the LSS group, indicating that the increase in CA and loading in the forefoot of the patients with LSS after the occurrence of NIC could also be caused by the patient's center of gravity moving to the anterior direction. This corresponded to the CT% result. It was also found that in the LSS group, the loading on the lateral feet increased and the CA increased after the occurrence of NIC compared with those before NIC in the LSS group. This study considered that patients with LSS also had a medial-lateral direction balance disorder. SaSaKi et al. [11] studied the characteristics of postural sway during quiet standing before and after the occurrence of NIC. In addition, they found that the postural sway in the left and right directions of patients with LSS increased after the occurrence of NIC, and the center of pressure shifted to the symptomatic side, implying the patient's risk of falling to the symptomatic side. Yoshio et al. [18] analyzed the gait analysis of patients with LSS and showed that patients with more severe symptoms of LSS presented a significantly higher swing index reflecting the left-right balance function compared with ordinary subjects. This might be because the symptoms of lower limbs caused by intravertebral compression could affect the load capacity of the affected limbs. In this study, the plantar pressure data of the symptomatic side or the more severe side were selected for analysis. During the entire stance phase, the patient suffered from insufficient muscle strength in the lower limbs due to the weakness, pain, or numbness of the symptomatic side. The body's center of gravity could be further biased to the symptomatic side, resulting in the transfer of plantar pressure distribution to the lateral side of the symptomatic foot.

Impaired balance in patients with LSS has been demonstrated in multiple studies. Aleksandra et al. [29] and Elisabeth [30] et al. conducted studies on the static and dynamic balance functions of patients with LSS, respectively, and confirmed that these patients had a higher risk of falling compared with ordinary subjects. Based on the aforementioned studies, the causes of changes in the gait cycle and plantar pressure distribution in patients with LSS were further analyzed in this study. Before the occurrence of NIC, patients with LSS maintained a mild lumbar forward flexion posture, and the center of gravity moved to the anterior direction. At this time, their body could maintain balance by adjusting the time distribution of each phase of the stance phase [21, 23, 31], and the plantar pressure distribution was close to that in normal subjects. However, these changes caused pressure to shift to the anterior and medial directions due to the “out-toe” gait [28]. After the occurrence of NIC, the patient further flexed the lumbar spine forward to reduce the symptoms, causing the center of gravity to move more in the anterior direction. At this time, the adjustment of the time distribution of the stance phases in each subphase might not be enough to compensate, so the CA and loading of the hindfoot decreased. By relying on the forefoot to contact the ground, the forefoot could be more susceptible to suffer from overuse injuries or strain diseases [31]. The uneven plantar pressure distribution in the anterior and posterior directions also indicated that the patient was at risk of falling to the anterior direction [11, 21, 23]. In addition, the occurrence of NIC made the lower limb muscles on the side of the symptoms contract with weakness or numbness, pain, and other symptoms. Hence, patients with LSS could not maintain the center of gravity of their bodies. Therefore, they shifted the pressure distribution of the plantar from the medial side to the lateral side of the symptomatic side foot. At the same time, they had a risk of falling to the symptomatic side.

This study had some limitations. Only the plantar pressure analysis was performed on patients with L4–L5 level spinal canal stenosis, considering this to be more common in clinic [1, 2], compared with the other levels of stenosis. Further studies are needed to explore the effects of spinal stenosis in different segments on the plantar pressure distribution of patients. However, previous gait analysis was performed on the selected patients with L4–L5 level stenosis [11, 30, 31]. Therefore, patients with LSS with spinal stenosis of other levels or multiple segments were not included in the experimental group, that is, the sample size was small. Future studies should use large samples and involve a comprehensive analysis. Second, only the symptomatic side or the more severe side foot was selected for analysis, which was not conducive to the overall assessment of the walking and balance functions of patients. Moreover, after the development of symptoms of NIC in patients with LSS, the patients were not asked to continue walking until they could not, which otherwise would have helped better understand NIC. Finally, the relation of the severity of NIC to the distribution of plantar pressure in patients with LSS was not evaluated in this study. This is a promising research direction, which can help create an objective and quantitative symptom assessment method to apply in clinic.

## 5. Conclusions

In summary, plantar pressure tests were conducted before and after the occurrence of NIC in patients with LSS, and the results were compared with those in healthy adults in this study. The results revealed the following differences in the distribution of plantar pressure: (1) patients with LSS could present the posture of the lumbar forward flexion when walking. As a result, the center of gravity moved in the anterior direction compared with the healthy subjects, increasing the CT and loading of the forefoot. The forefoot might have a higher risk of strain disease or overuse injury. (2) The occurrence of NIC could affect the plantar pressure distribution of the patients with LSS, predicting the patient's risk of falling to the anterior direction and to the symptomatic side. Future gait analysis studies should focus on investigating the changes in plantar pressure distribution after the occurrence of NIC in patients with LSS and correlate them with patients' walking functions, such as the maximum walking distance or the level of balance function. It is promising to provide a new objectively method for evaluating the walking capabilities of patients with LSS.

## Figures and Tables

**Table 1:** Comparison of the main demographic parameters in LSS group and control group.

Items	LSS group	Control group	*P* value
Age	60.00 ± 7.27	59.33 ± 7.40	0.755
Height (cm)	168.50 ± 7.48	169.25 ± 8.54	0.932
Weight (kg)	71.58 ± 7.01	72.50 ± 9.10	0.977
Foot length (mm)	252.50 ± 16.45	255.00 ± 17.19	0.843
BMI (kg/m^2^)	25.18 ± 1.02	25.22 ± 1.15	0.843
T-score	−0.33 ± 0.07	−0.27 ± 0.19	0.932

**Table 2:** Comparison of CT% in the four sub-stance phases and total CT of the foot for each group.

Stance phases	CT%/%				*P*			
	LSS		Control		P_s_		P_g_	
	1^st^	2^nd^	1^st^	2^nd^	LSS	Control	1^st^	2^nd^
ICP	7.65 ± 2.16	2.86 ± 1.94	9.68 ± 2.11	9.44 ± 1.89	0.002∗	0.158	0.028∗	<0.001∗
FFCP	5.94 ± 1.72	2.49 ± 1.37	6.36 ± 1.65	6.20 ± 1.78	0.002∗	0.272	0.478	<0.001∗
FFP	45.96 ± 3.22	49.36 ± 2.96	46.28 ± 3.15	46.54 ± 2.61	0.002∗	0.146	0.671	0.033∗
FFPOP	40.45 ± 3.18	45.30 ± 3.27	37.76 ± 3.02	37.82 ± 2.60	0.002∗	0.875	0.033∗	<0.001∗
CT/ms	755.66 ± 53.08	858.23 ± 56.40	692.61 ± 44.77	692.02 ± 46.52	0.002∗	0.754	0.003∗	<0.001∗

Values are expressed as means ± standard deviation. Pg: comparisons between the LSS group and the control group using independent sample t test; Ps: comparisons between the 2 tests in the LSS group and control group using paired t test. 1^st^: first test. 2^nd^: second test. CT%: contact time % (contact time of the 4 sub-phases normalized to the stance time of single leg); FFCP: forefoot contact phase; FFP: foot flat phase; FFPOP: forefoot push off phase; ICP: initial contact phase. ∗*P* < 0.05.

**Table 3:** Comparison of FPA for each group.

FPA/°				*P*			
LSS		LSS		P_s_		P_g_	
1^st^	2^nd^	1^st^	2^nd^	LSS	Control	1^st^	2^nd^
22.96 ± 5.39	23.09 ± 5.33	9.91 ± 3.50	9.85 ± 3.25	0.723	0.906	<0.001∗	<0.001∗

Values are expressed as means ± standard deviation. Pg: comparisons between the LSS group and the control group using independent sample *t*-test; Ps: comparisons between the 2 tests in the LSS group and control group using paired *t*-test. 1^st^: first test. 2^nd^: second test. FPA: foot progression angle. ∗*P* < 0.05.

**Table 4:** Comparison of the PTI in various zones of the foot for each group.

Foot zones	PTI/(N·s·cm^−2^)				*P*			
	LSS		Control		P_s_		P_g_	
	1^st^	2^nd^	1^st^	2^nd^	LSS	Control	1^st^	2^nd^
T1	1.25 ± 0.64	1.27 ± 0.56	1.22 ± 0.83	1.30 ± 0.80	0.785	0.234	0.478	0.755
T2~5	0.24 ± 0.13	0.31 ± 0.16	0.20 ± 0.10	0.23 ± 0.09	0.198	0.317	0.478	0.101
M1	3.18 ± 1.12	4.53 ± 1.20	2.20 ± 0.91	2.21 ± 0.70	0.002∗	0.633	0.045∗	<0.001∗
M2	7.41 ± 1.66	8.72 ± 1.66	6.12 ± 1.66	5.98 ± 1.69	0.002∗	0.251	0.033∗	<0.001∗
M3	10.23 ± 2.62	11.49 ± 2.45	9.22 ± 3.13	8.83 ± 2.67	0.013∗	0.142	0.077	0.001∗
M4	5.85 ± 2.08	7.75 ± 1.77	5.27 ± 2.12	5.41 ± 1.97	0.002∗	0.325	0.378	0.004∗
M5	2.58 ± 1.54	2.63 ± 1.64	2.32 ± 1.19	2.35 ± 1.50	0.385	0.478	0.671	0.713
MF	1.06 ± 0.38	0.84 ± 0.40	0.87 ± 0.41	0.97 ± 0.46	0.090	0.277	0.266	0.514
MH	4.43 ± 1.22	1.92 ± 1.10	4.20 ± 1.36	4.38 ± 1.47	0.002∗	0.083	0.590	<0.001∗
LH	3.50 ± 0.94	1.83 ± 0.94	3.65 ± 0.89	3.45 ± 1.07	0.002∗	0.058	0.630	0.001

Values are expressed as means ± standard deviation. Pg: comparisons between the LSS group and the control group using independent sample *t*-test; Ps: comparisons between the 2 tests in the LSS group and control group using paired *t*-test. PTI: pressure-time integral. 1^st^: first test. 2^nd^: second test. T1: hallux. T2–5: toes 2–5. M1: first metatarsal. M2: second metatarsal. M3: third metatarsal. M4: fourth metatarsal. M5: fifth metatarsal. MF: midfoot. LH: lateral heel. MH: medial heel. ∗*P* < 0.05.

**Table 5:** Comparison of CA% in various zones of the foot for each group.

Foot zones	CA%/%				*P*			
	LSS		Control		P_s_		P_g_	
	1^st^	2^nd^	1^st^	2^nd^	LSS	Control	1^st^	2^nd^
T1	9.99 ± 1.11	10.88 ± 1.17	9.98 ± 1.42	10.06 ± 1.08	0.002∗	0.583	0.630	0.039∗
T2~5	9.42 ± 1.43	10.37 ± 1.57	8.67 ± 1.85	8.76 ± 1.64	0.002∗	0.388	0.319	0.039∗
M1	7.62 ± 1.03	8.91 ± 1.02	6.64 ± 1.04	6.63 ± 0.99	0.002∗	0.894	0.021∗	0.002∗
M2	8.05 ± 0.64	8.76 ± 0.86	7.19 ± 1.01	7.09 ± 0.88	0.010∗	0.239	0.017∗	0.001∗
M3	7.17 ± 0.75	8.19 ± 0.76	6.66 ± 0.70	6.80 ± 0.73	0.002∗	0.099	0.671	<0.001∗
M4	5.66 ± 1.12	6.70 ± 0.98	5.84 ± 0.77	5.88 ± 0.77	0.002∗	0.875	0.178	0.012∗
M5	6.64 ± 1.38	6.78 ± 1.52	7.07 ± 1.33	7.11 ± 1.12	0.084	0.937	0.319	0.932
MF	23.47 ± 1.88	23.88 ± 1.59	24.29 ± 3.13	24.76 ± 2.53	0.724	0.136	0.160	0.068
MH	12.22 ± 1.92	8.20 ± 1.73	12.68 ± 1.47	12.11 ± 0.85	0.002∗	0.060	0.410	<0.001∗
LH	9.97 ± 1.44	7.35 ± 1.98	10.99 ± 1.01	10.80 ± 1.14	0.003∗	0.424	0.060	<0.001∗

Values are expressed as means ± standard deviation. Pg: comparisons between the LSS group and the control group using independent sample *t*-test; Ps: comparisons between the 2 tests in the LSS group and control group using paired *t*-test. CA%: contact area % (contact area of the 10 foot zones normalized to the whole contact area of the foot). 1^st^: first test. 2^nd^: second test. T1: hallux. T2–5: toes 2–5. M1: first metatarsal. M2: second metatarsal. M3: third metatarsal. M4: fourth metatarsal. M5: fifth metatarsal. MF: midfoot. LH: lateral heel. MH: medial heel. ∗*P* < 0.05.

## Data Availability

The authors declare all data are fully available without restriction.
